# Characteristics and clinical management of patients admitted to cholera wards in a regional referral hospital during the 2012 epidemic in Sierra Leone

**DOI:** 10.3402/gha.v8.25266

**Published:** 2015-01-06

**Authors:** Alexander Blacklock, Andrew Sesay, Abdul Kamara, Mamud Kamara, Claire Blacklock

**Affiliations:** 1 Makeni Government Hospital, Bombali District, Sierra Leone; 2 Binkolo Peripheral Health Unit, Bombali District, Sierra Leone; 3 Central Public Health Reference Laboratory, Ministry of Health and Sanitation, Freetown, Sierra Leone; 4 Nuffield Department of Primary Care Health Sciences, University of Oxford, Oxford, United Kingdom

**Keywords:** cholera, triage, dehydration, epidemic, Sierra Leone

## Abstract

**Background and objectives:**

In 2012, Sierra Leone suffered a nationwide cholera epidemic which affected the capital Freetown and also the provinces. This study aims to describe the characteristics and clinical management of patients admitted to cholera isolation wards of the main referral hospital in the Northern Province and compare management with standard guidelines.

**Design:**

All available clinical records of patients from the cholera isolation wards were reviewed retrospectively. There was no active case finding. The following data were collected from the clinical records after patients had left the ward: date of admission, demographics, symptoms, dehydration status, diagnoses, tests and treatments given, length of stay, and outcomes.

**Results:**

A total of 798 patients were admitted, of whom 443 (55.5%) were female. There were 18 deaths (2.3%). Assessment of dehydration status was recorded in 517 (64.8%) of clinical records. An alternative or additional diagnosis was made for 214 patients (26.8%). Intravenous (IV) fluids were prescribed to 767 patients (96.1%), including 95% of 141 patients who had documentation of being not severely dehydrated. A history of vomiting was documented in 92.1% of all patients. Oral rehydration solution (ORS) was given to 629 (78.8%) patients. Doxycycline was given to 380 (47.6%) patients, erythromycin to 34 (4.3%), and other antibiotics were used on 247 occasions. Zinc was given to 209 (26.2%).

**Discussion:**

This retrospective study highlights the need for efforts to improve the quality of triage, adherence to clinical guidance, and record keeping.

**Conclusions:**

Data collection and analysis of clinical practices during an epidemic situation would enable faster identification of those areas requiring intervention and improvement.

Cholera (caused by *Vibrio cholerae*) remains a major global public health challenge especially in situations where the spread of disease is exacerbated by poor water supply, hygiene, and sanitation (1). Cases of cholera (*n*=245,393) were reported from 48 different countries in 2012 (2).

In the rainy season of 2012, Sierra Leone suffered its largest ever cholera epidemic, affecting the capital Freetown (52% of all reported cases were from the Western Area) and all but one of its other districts. The total number of reported cases was 22,885, with 298 reported deaths (3). The World Health Organization (WHO) and international humanitarian organisations such as Médicins Sans Frontières and the International Red Cross assisted Government efforts to control the epidemic. The Government of Sierra Leone committed to providing treatment for cholera free of charge in all Government health facilities. Prior to 2012, Sierra Leone had witnessed a series of smaller outbreaks, the most recent in 2008 with 62 reported cases (3).

Makeni Government Hospital is one of three Regional Referral Hospitals in Sierra Leone outside Freetown. During the outbreak, the hospital established two designated cholera isolation wards (male and female). Patients were admitted to the cholera isolation wards following initial presumptive clinical diagnosis and triage in outpatients, or later from a specially erected triage tent, where initial medication and fluid management were also prescribed. Medical ward rounds of inpatients were conducted. A clinical diagnosis of cholera was used after initial small-scale use of RDTs (rapid diagnostic tests).

Epidemics of cholera have been associated with high morbidity and significant mortality (4). Mortality is reduced by prompt rehydration and access to health facilities (5). However, for common endemic diseases (e.g. malaria, pneumonia), previous studies have shown examples of poor adherence to clinical protocols and treatment guidelines in constrained health settings (6, 7). Standard guidelines for the treatment of cholera and dehydration are summarised in Table 1 (8, 9).

**Table 1:** Standard guidelines for the assessment and management of dehydration

Classification of dehydration	Clinical features	Treatment advised
None	None of the features listed below	ORS
Some	Two or more of the following: Sunken eyes Absence of tears Dry mouth and tongue Thirsty and drinking eagerly Skin pinch goes back slowly (child – restless/irritable)	Rehydration with ORS and monitor patient
Severe	In addition to the above: Lethargic, unconscious or floppy Unable to drink (or drinking poorly) Radial pulse weak Skin pinch goes back very slowly	Rehydration with IV fluids, then with ORS when dehydration no longer severe

This retrospective notes review aims to describe the characteristics and clinical management of patients admitted to cholera isolation wards of the main referral hospital in the Northern Province and compare management with standard guidelines.

## Design

### Study design and setting

A retrospective clinical records review was undertaken in the Northern Provincial Government referral hospital, Sierra Leone.

### Data collection

All available clinical records were reviewed for adult and paediatric admissions to the male and female cholera isolation wards. There was no active case finding. Clinical records were reviewed by two UK-trained general practitioners who had been working as doctors on the cholera wards during the epidemic. Both doctors had been working at the hospital for 1 month before the start of the epidemic. From each clinical record, the following data were retrospectively extracted: date of admission, patient demographics (sex, age in years), symptoms, documentation of dehydration status, additional diagnoses, tests undertaken, treatment given, length of stay (in days), and outcome of admission (discharged, died, left hospital without being formally discharged).

‘Admission’ was defined as any patient who had entered the cholera isolation wards following outpatient triage, instead of being managed and discharged directly from the outpatient department. This was regardless of subsequent length of stay on the ward.

‘Length of stay’ was defined as the total number of days on which the patient was documented to have been present on the isolation wards. This was calculated using the date of discharge or death, or the date on which the person left the ward without formal discharge, which was calculated using the last day that any documentation had been made in the patient's notes or drug chart.

‘Documentation of dehydration status’ was defined as any text recorded in the patient's clinical record, either at triage or ward review, considered to be relating to assessment of severity of dehydration. Two doctors who had been working on the cholera wards reached consensus on specific words or phrases that indicated assessment of dehydration status (see Supplementary Table A for examples of words and phrases).

A separate analysis of rehydration management was performed for: 1) those cases for whom severe dehydration had been clearly documented in the patient record (i.e. documented as severe, or clinical features of severe dehydration clearly documented), and 2) for cases for whom not severely dehydrated had been clearly documented (i.e. documented as mild, or not too/very dehydrated). Cases where insufficient signs had been recorded to be able to categorise dehydration as severe or not severe, or where dehydration had been recorded as moderate, were not included in this sub-analysis.

‘Clinical diagnoses other than cholera’ were defined as any one of: 1) a clinical diagnosis other than cholera documented by a health care professional in the notes, 2) a diagnostic test result, for example, positive thick film in the case of malaria, or 3) diagnosis-specific treatment prescribed [e.g. Artesunate Combination Therapy (ACT) for malaria]. We did not therefore limit diagnoses to those for which confirmatory laboratory tests were positive.

### Analysis

Data were entered into Microsoft Excel (2007 version), and SPSS (version 21.0) was used for analysis of frequencies. Figures were constructed using Microsoft Excel (2007 version).

## Results

**Fig. 1:**
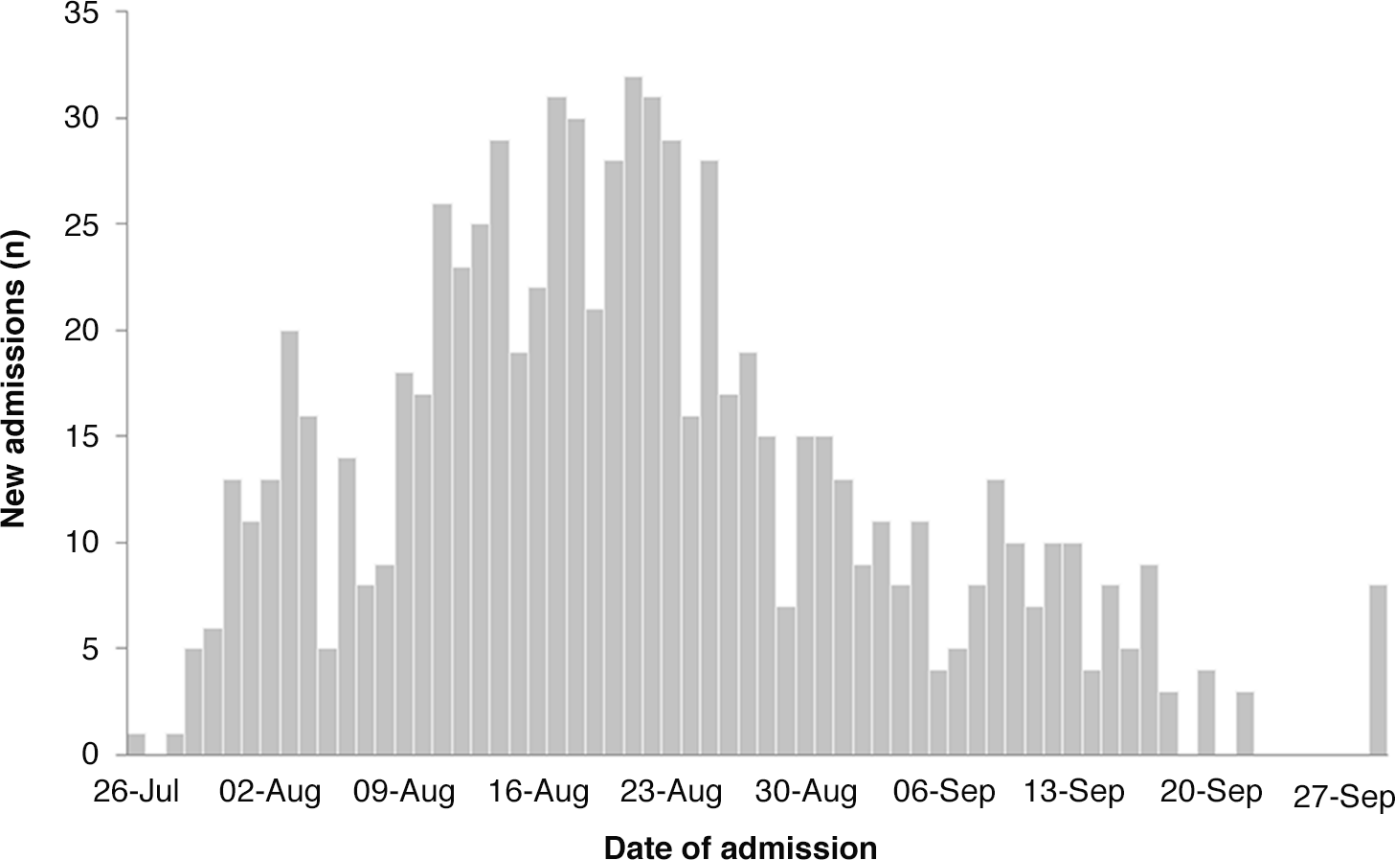
New admissions to the male and female cholera wards during the epidemic period.

**Fig. 2:**
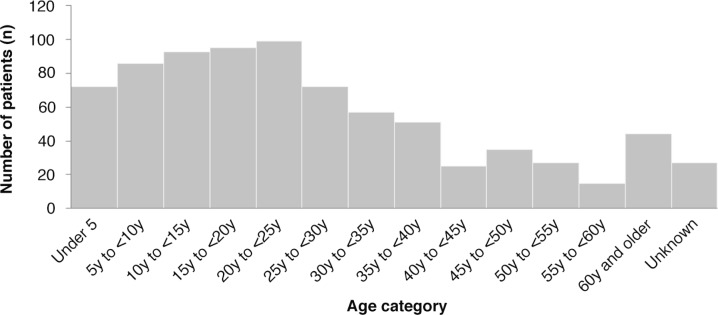
Age distribution of total patient population admitted to the cholera wards.

A total of 798 patients were admitted to the cholera isolation wards during the epidemic period between 26 July 2012 and 22 September 2012. The number of new admissions peaked towards the end of August 2012, with the highest number on 21 August (*n*=31) (Fig. 1). During the epidemic period reported, there were 18 deaths on the isolation wards (2.3%). Of the patients that died, one had laboratory confirmed cholera (RDT positive), and RDTs were unavailable for the remaining patients. Three of the deceased patients were diagnosed with typhoid. Of the patients admitted to the cholera wards, 443 (55.5%) were female and 594 (74.4%) were aged less than 35 years. Children under-5 years of age accounted for 72, 12% of those were aged less than 35 years. Elderly patients (over 60 years), accounted for just 44 cases (5.5%) (Fig. 2). Length of stay in hospital was generally short, with 569 patients (71.3%) remaining on the wards for 2 days or less. In addition, 304 patients (38.1%) left the ward before being formally discharged.

Assessment relating to dehydration status was recorded in the clinical notes of 517 (64.8%) patients. There was no documentation of any symptoms or signs relating to dehydration status for the remaining 35.2% of patients. Of patients admitted to the cholera wards, 214 patients (26.8%) received a combined total of 282 additional or alternative clinical diagnoses of malaria, typhoid, dysentery, or ‘other’ (Table 2 and Supplementary Table B).

**Table 2:** Additional or alternative diagnoses given to patients admitted to cholera wards

Diagnosis given	Frequency of diagnosis (*n*)	Percentage of total admissions
Malaria	122	15.3
Dysentery	12	1.5
Typhoid	106	13.3
Other	42	5.3

A total of 15 cholera RDT results were available from the clinical records. Of these, 4 were positive for cholera (26.7%) and 11 were negative. A further 13 RDTs were documented as requested in the patient notes, but results were unavailable. A blood film for malaria parasites was requested for 178 patients, of which 51 blood films were positive for malaria parasites, 94 were negative, and the results were unavailable for the remaining 33 patients. Widal testing for typhoid was requested for 96 patients, and was reactive in 62 cases, non-reactive in 22 cases, and the result unavailable in 12 cases.

Treatment given to patients admitted to the cholera wards is summarised in Table 3. Almost all patients admitted to the cholera wards received intravenous (IV) fluids (*n*=767, 96.1%), and just 629 (78.8%) received oral rehydration solution (ORS). Of the 80 patients for whom there was clear documentation of severe dehydration in the clinic record, all received IV fluids and 74 (92.5%) also received ORS. The notes of 141 (17.7%) patients indicated that there was either no dehydration or mild dehydration, at triage or at ward review. Of these patients, 134 (95%) received IV fluids and 126 (89.4%) received ORS. It is possible that some of these patients received initial IV rehydration before the documentation of dehydration had been made in the clinical record. However, it is still unlikely that severe dehydration was initially present in the majority of these cases. A history of vomiting was present for almost all cases (735 cases, 92.1%), including for 131 patients (92.9%) with documentation of non-severe dehydration. Doxycycline was given to 380 patients (47.6%), including to 19/191 patients under 12 years. Erythromycin was given to 34 patients (4.3%), including to 8/191 patients under 12 years. Zinc was given to 209 patients (26.2%), including to 128/191 children under 12 years. Other antibiotics were used on 247 occasions (see Table 3 for details). Combination treatment with doxycycline or erythromycin plus another antibiotic was given to 84 patients (10.5%). ACT was prescribed to 114 patients (14.3%) (Table 3).

**Table 3:** Treatments given to patients admitted to cholera wards

Treatment given	Frequency (*n*)	Percentage of patients treated
Intravenous fluids	767	96.1
Oral rehydration solution	629	78.8
Doxycycline	380	47.6
Zinc	209	26.2
Ciprofloxacin	115	14.4
Artesunate combination therapy	114	14.3
Metronidazole	66	8.3
Paracetamol	50	6.3
Ampicillin/amoxicillin	42	5.3
Antiemetic	35	4.4
Erythromycin	34	4.3
Quinine	19	2.4
Chloramphenicol	16	2.4
Omeprazole	15	2.4
Potassium	13	0.9
Artemether	7	0.9
Gentamicin	5	0.6
Cefixime	2	0.3
Benzathine benzylpenicillin	1	0.1
Other	11	1.4

## Discussion

The Northern Provincial Government Hospital admitted and treated 798 patients on its cholera isolation wards, between 26th July and 27th September during the cholera epidemic in Sierra Leone in 2012. These admissions show an epidemic pattern.

More women than men were admitted. This might reflect gender differences in domestic tasks and associated risks of cholera transmission or baseline health status. This finding is in keeping with studies in other settings (1, 10). Most admissions were in those under 35 years of age. This could reflect differences in risk across age groups, or it could simply reflect the age distribution of the population at large.

Many patients were treated for additional diagnoses such as malaria during their admission, which can also present with symptoms of gastroenteritis. Confirmatory diagnostic tests were not available for all such patients; therefore, some were treated on clinical suspicion alone. This raises the issue whether to admit such patients to cholera isolation wards and increase the risk of potentially acquiring cholera or to presumptively contain all patients with symptoms of gastroenteritis in order to protect others on the general wards. Improved initial triage might therefore enhance infection control.

Almost all patients admitted received IV fluids. For many of these patients, dehydration status was not documented. It is possible that these patients had severe dehydration, but for at least 17.7% of patients for whom there was documentation that they were not severely dehydrated at triage or ward review, this does not appear to be the case. In almost all cases however there was a history of vomiting, which may have influenced the decision to treat with IV fluids (11), and some patients may have received IV fluids before dehydration status was documented in the clinical record. ORS was less frequently prescribed than IV fluids for patients without severe dehydration, out of line with guidance (8, 9). It is therefore a lesson for future diarrhoeal epidemic situations that initial triage and assessment of dehydration severity is of high importance, and impacts both on individual patient treatment, and on managing finite resources. Supply issues during the epidemic may have played a role, however the need for enhanced training and support in the systematic structured use of the WHO Treatment Plans A, B and C for dehydration in managing such patients is highlighted (8, 9, 12, 13). The challenge of addressing health beliefs held by patients (e.g. pressure on health staff to give parenteral treatments) should also be recognised.

Variation in prescribing practices was found during the study period, including variation in antibiotic choice. In some cases antibiotics were used to treat alternative or additional clinical diagnoses, such as typhoid, respiratory tract infection, or dysentery, but variation was also seen in prescribed medicines for suspected cholera. For example, metronidazole was given to 8.3% of all admitted cases, whereas only 1.5% of cases were documented to have dysentery. This suggests that metronidazole was sometimes used as a treatment for suspected cholera, despite not being recommended (9). Whilst effective antibiotic therapy given in severe cases is known to reduce both the volume and duration of diarrhoea (10), there is concern about resistant strains (4, 14, 15) and the need for clear and focused antibiotic treatment. This emphasises the need for structured adherence to protocol-led service delivery in epidemic situations to reduce the risk of emerging antibiotic resistance as well as individual patient cost when required to purchase drugs.

### Strengths of the study

This is the first study of a cholera epidemic from the Northern Province in Sierra Leone. All available patient records from the cholera isolation wards were included in the study. This study is among few that focus on aspects of clinical diagnosis and clinical management of patients during a cholera epidemic.

### Limitations of the study

The data set does not include those patients who were not admitted to the ward from outpatients. We are therefore unable to assess the full impact of outpatient triage. We did not include patients in the community who did not or could not attend the hospital for treatment. This therefore biases our findings to just those patients who were able to access government healthcare, so does not estimate the magnitude or severity of the epidemic in Northern Province, Sierra Leone. The outcome of those patients who left before formal discharge is unknown; however, anecdotal recollection supports the presumption that the vast majority of these had clinically improved and did not want to stay to see a doctor. An unknown number of patient records were not included in the dataset due to being mislaid from the wards. We were unable to assess the impact of supply on choice of treatment. A few patient records were noted to be incomplete during data collection (e.g. the drug chart was missing), so only available data from the remaining record were used. Missing clinical records, and incomplete records, will have affected the quality of the data collected. Training for future epidemic situations should also focus on the importance of accurate clinical record keeping and effective data collection, to improve monitoring and to guide appropriate quality improvement efforts.

## Conclusions

This retrospective study highlights the need for efforts to monitor and improve the quality of triage, adherence to clinical guidance, and record keeping during an epidemic. Data collection and analysis of clinical practices during an epidemic situation could allow faster identification of areas for improvement and allow these to be addressed in real time.
